# Left Atrial Function after Atrial Fibrillation Cryoablation Concomitant to Minimally Invasive Mitral Valve Repair: A Pilot Study on Long-Term Results and Clinical Implications

**DOI:** 10.3390/medicina55100709

**Published:** 2019-10-21

**Authors:** Matteo Anselmino, Chiara Rovera, Giovanni Marchetto, Davide Castagno, Mara Morello, Simone Frea, Fiorenzo Gaita, Mauro Rinaldi, Gaetano Maria De Ferrari

**Affiliations:** 1 Division of Cardiology, “Città della Salute e della Scienza di Torino” Hospital, Department of Medical Sciences, University of Turin, 10124 Torino, Italy; davide.castagno@unito.it (D.C.); mara.morello@unito.it (M.M.); frea.simone@gmail.com (S.F.); gaetanomaria.deferrari@unito.it (G.M.D.F.); 2 Cardiology Unit, “Ospedale Civico”, Chivasso, 10034 Torino, Italy; roverachiara@gmail.com; 3 Division of Cardiac Surgery, “Città della Salute e della Scienza di Torino” Hospital, University of Turin, 10124 Torino, Italy; giovanni.marchetto@libero.it (G.M.); mauro.rinaldi@unito.it (M.R.); 4 Cardiology Department, Clinica Pinna Pintor, 10129 Torino, Italy; fiorenzo.gaita@unito.it

**Keywords:** atrial fibrillation, surgical cryoablation, left atrial function, minimally invasive mitral valve repair, echocardiography, ischemic cerebral events

## Abstract

*Background and Objectives:* Surgical atrial fibrillation (AF) ablation concomitant to minimally invasive mitral valve repair has been proven to offer improved short- and long-term sinus rhythm (SR) maintenance compared to mitral valve surgery only. The objective of the present study was to explore, by thorough echocardiographic assessment, long-term morphological and functional left atrial (LA) outcomes after this combined surgical procedure. *Materials and Methods*: From October 2006 to November 2015, 48 patients underwent minimally invasive mitral valve repair and concomitant surgical AF cryoablation. *Results:* After 3.8 ± 2.2 years, 30 (71.4%) of those completing the follow-up (*n* = 42, 87.5%) presented SR. During follow-up, four (9.5%) patients suffered from cerebrovascular accidents and two of these subjects had a long-standing persistent AF relapse and were in AF at the time of the event, while the other two were in SR. An echocardiographic study focused on LA characteristics was performed in 29 patients (69.0%). Atrial morphology and function (e.g., maximal LA volume indexed to body surface area and total LA emptying fraction derived from volumes) in patients with stable SR (60.6 ± 13.1 mL/mq and 25.1 ± 7.3%) were significantly better than in those with AF relapses (76.8 ± 16.2 mL/mq and 17.5 ± 7.4%; respectively, *p* = 0.008 and *p* = 0.015). At follow-up, patients who suffered from ischemic cerebral events had maximal LA volume indexed to body surface area 61 ± 17.8 mL/mq, with total LA emptying fraction derived from volumes 23.6 ± 13.7%; patients with strokes in SR showed very enlarged LA volume (>70 mL/mq). *Conclusions*: AF cryoablation concomitant with minimally invasive mitral valve repair provides a high rate of SR maintenance and this relates to improved long-term morphological and functional LA outcomes. Further prospective studies are needed to define the cut-off values determining an increase in the risk for thromboembolic complications in patients with restored stable SR.

## 1. Introduction

Atrial fibrillation (AF) prevalence in patients with indication for mitral valve surgery is about 30–54% [1], it has a strong impact on hemodynamics [2], and it has been demonstrated to significantly affect the mortality rate [3,4].

Concomitant surgical AF ablation to video-assisted minimally invasive mitral valve surgery (MIMVS) [5,6,7] has been proven to offer improved short- and long-term sinus rhythm (SR) maintenance compared to patients undergoing mitral valve surgery only (73% versus 43% of SR maintenance at 12 months’ follow-up) [8] without increasing complications [9,10].

Therefore, SR maintenance seems achievable, but does this reflect in an improved atrial function? It has been demonstrated that SR maintenance is related to reduced left atrial volumes [11], but little is known about left atrial (LA) functional properties in this clinical setting. In fact, an organized atrial activity is not always accompanied by an effective mechanical atrial contraction [12].

In patients with underlying mitral valve diseases, atrial remodeling is remarkable: longstanding volume overload to the LA results in chronic stretching and atrophy of atrial myocytes, interstitial fibrosis, overall thinning, and dilatation of the LA wall, which may relate to functional alterations despite the underling electrical activity.

To date, however, it remains unknown to which degree “the residual atrial function” after a successful AF ablation predicts clinical outcome. For example, which level of contractility is required to avoid the increased thromboembolic risk of a “static” LA, despite SR?

The aim of the present study was therefore to describe, by a thorough echocardiographic assessment, long term LA morphology and function in patients submitted to surgical ablation of persistent/long-term persistent AF concomitant to MIMVS.

## 2. Materials and Methods

### 2.1. Surgical Procedure

According to current guidelines, patients referred to our Cardiac Surgery Division for mitral valve disease and AF resistant to antiarrhythmic therapy, when technically feasible, were proposed video-assisted MIMVS via right mini-thoracotomy through the fourth intercostal space and concomitant left sided AF cryoablation. All enrolled patients were retrospectively identified, starting since October 2006. Patients that had already performed a cardiac surgical procedure or a previous transcatheter AF ablation were not included in this series. Each patient in our study signed a written informed consent for inclusion. The study was conducted in accordance with the Declaration of Helsinki, was observational and retrospective, did not add treatment or modify conventional surgical procedure for the specific clinical indication and was approved by the local Institutional Review Board (Project Identification “CryoMIMS—Concomitant Cryoablation to Video-assisted Minimally INvasive Mitral Valve Surgery”, code 9718; date of approval 1 February 2016).

Surgical technique has already been described [13].

Concomitant left sided AF cryoablation (Argon based Cryomaze, Cryoflex Medtronic, Minneapolis, MN, USA) consisted of isolation of the pulmonary veins (PVs) and of the posterior LA wall between the veins by a “U” encircling cryolesion connected to the surgical paraseptal LA incision performed for mitral exposure, eventually creating the so-called “box lesion”. In addition, a linear cryolesion was performed from the previously created box lesion to the mitral valve annulus to block conduction across the left atrial isthmus (“mitral line”) [6,14]. The surgical procedure performed was the same in every patient included in the study.

### 2.2. Echocardiographic Analysis

Complete echocardiographic assessment was performed in patients undergoing mitral valve repair by the MIMVS approach and concomitant left sided AF cryoablation. The echocardiographic assessment was performed at 4.0 ± 2.1 years after the surgical procedure. Subjects who underwent mitral valve replacement with biological or mechanical prosthesis were excluded due to distortions related to the presence of the prosthetic scaffold.

**Figure 1:**
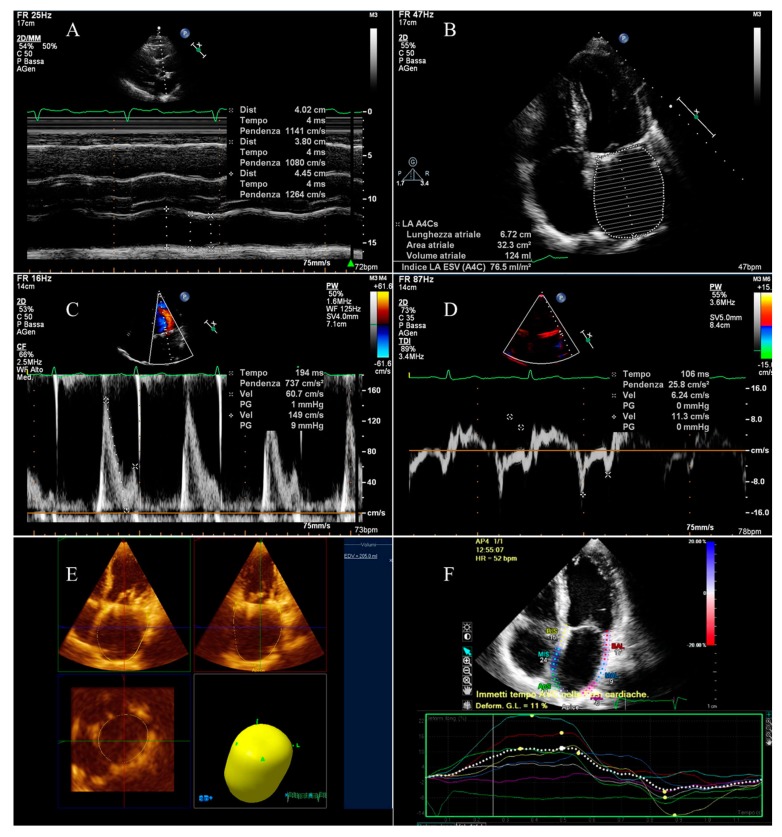
Echocardiographic evaluation of left atrial function: (**A**) measurement of antero-posterior diameters; (**B**) evaluations of areas and volumes and measurement of maximum supero-inferior diameter; (**C**) measurement of transmitral peak velocity of the late filling wave (A wave); (**D**) measurement of lateral mitral annulus peak velocity related to atrial contraction (a’ wave) and evaluation of atrial conduction delay; (**E**) calculation of maximum 3D volume; (**F**) evaluation of global longitudinal atrial strain.

Echocardiographic scans were performed by a Philips ultrasound system (iE33 xMATRIX, Andover, MA, USA) with S5-1 sector array probe and X3-1 3D probe. All the examinations were carried out by the same operator. Standard measurements were computed based on the American Society of Echocardiography guidelines. Maximum left atrial diameter (LAD max), minimum (LAD min) and at the beginning of atrial contraction (LAD pre-A) antero-posterior diameters were measured in the parasternal long axis view by either B-mode or M-mode technique (Figure 1A).

The apical four chamber view was used to measure maximum left atrial supero-inferior diameter (Figure 1B).

The left atrial volumes were evaluated by modified Simpson’s method using the apical four chamber view at the tele-systolic frame preceding the mitral valve opening (maximum volume, LAV max), at the tele-diastolic frame preceding mitral valve closure (minimum volume, LAV min), and at the beginning of the P wave for the subjects not in AF at the moment of the exam (pre A volume, LAV pre-A) (Figure 1B).

Volumes were subsequently indexed on the body surface area.

From the above-mentioned linear measurements and volumes, the following parameters defining different components of LA function were calculated:

- Left atrial active emptying fraction, related to the LA booster pump function;
- Left atrial passive emptying fraction, describing the LA conduit function;
- Left atrial total emptying fraction, defining the LA reservoir function.

Similarly, the LA areas (maximal, minimal, and at the beginning of the P wave) were measured (Figure 1B). The LA ejection fraction was calculated from maximal and minimal areas, being reduced if ≤45% [15].

Left atrial 3D maximum volume was calculated using the QLAB-3D Quantification (3DQ) Advanced application (Figure 1E).

The transmitral flow velocity was measured by pulsed Doppler echocardiography. Peak velocity of the early filling wave (E wave) and of the late filling wave (A wave) were determined (Figure 1C), considering a peak A wave velocity ≥10 cm/s an indicator of the presence of some atrial contraction [16].

The lateral and septal mitral annulus peak velocities related to early relaxation (e’ wave) and to atrial contraction (a’ wave) were evaluated by tissue Doppler imaging (TDI, Figure 1D). A TDI a’ ≤7 cm/s was considered an index of anomalous LA active contractile function [17].

Furthermore, atrial conduction time was measured: the PA-TDI interval was calculated, defined as the time interval between the beginning of the P wave and the TDI a’ wave (Figure 1D). The standard deviation of PA-TDI measurements performed on all segments was also calculated for every single patient.

Eventually, LA global longitudinal strain by two-dimensional speckle tracking was calculated for every patient by QLAB CMQ Cardiac Motion Quantification (Figure 1F).

### 2.3. Clinical Follow-Up and Event Definition

After discharge patients were followed by outpatient visits, including clinical examinations and ECG at 3, 6, and 12 months and then yearly. At least once a year, 24 h ECG Holter monitoring was performed. In case of symptoms recurrence between follow-up visits, patients were reassessed by clinical examination, ECG, and Holter monitoring. Electrophysiological study and transcatheter AF ablation were performed when indicated. All patients in the study were followed for at least six months.

A blanking period of three months was considered; following this interval any AF episode, persistent or paroxysmal, was accounted as an event.

### 2.4. Statistical Analysis

Categorical variables are reported as counts and percentages, while continuous variables as means and standard deviations (SDs). Correlations between parameters and study groups were tested in cross tabulation tables by means of the Pearson Chi-Square or Fisher’s exact test and by one-way ANOVA, respectively, for categorical and continuous variables. McNemar’s test was used on paired categorical variables. Kaplan Meier curves were computed to describe AF free survival over time. A two-sided *p*-value < 0.05 was considered statistically significant; all analyses were performed on SPSS 20.0 (IBM Corp., Armonk, NY, USA).

## 3. Results

From October 2006 to November 2015, 48 patients were submitted to minimally invasive mitral valve repair and concomitant AF cryoablation. The baseline characteristics of the study population are listed in Table 1.

Twenty (41.7%) patients underwent simple valve repair while 28 (58.3%) were submitted to complex valve repair. Seven subjects (14.6%) received concomitant tricuspid valve surgery. Only one procedure was electively converted to full sternotomy (2.1%) due to unexpected severe pleural adhesions. Total clamp time was 99.5 ± 25.6 min. There was no reopening for any cause.

About one third of the study population (18, 37.5%) suffered AF relapses during hospitalization, while 39 (81.3%) patients were discharged in SR. One patient (2.1%) required PM implantation in the subacute phase. Early mortality was 2.1% (one patient died due to respiratory complications following cardiac arrest resuscitated in the ward).

**Figure 2:**
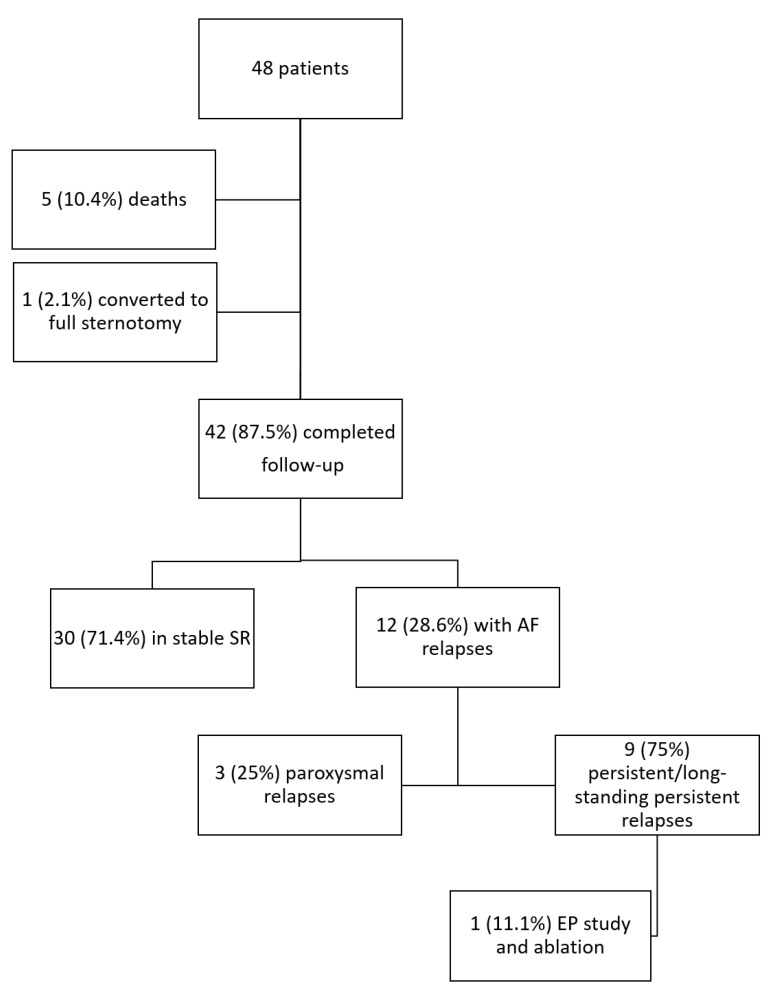
Study flow chart. AF: atrial fibrillation; EP: electrophysiological; SR: sinus rhythm.

By April 2016, 42 (87.5%) patients completed the follow-up after a mean of 3.8 ± 2.2 years from the procedure. The study flow chart is depicted in Figure 2.

Except the patient who died few days after surgery, the remaining four deaths were not due to cardiovascular causes.

In this time frame no patient required redo surgery.

NYHA functional class showed a significant improvement (NYHA ≥ 3 patients decreased from 64.6% pre-surgery to 4.8% at follow-up, *p* < 0.001). Three (7.1%) patients required PM implantation during follow-up.

Thirty patients (71.4%) maintained SR throughout the follow-up. Out of the 12 (28.6%) patients suffering AF relapses, three (25%) had paroxysmal episodes, while nine (75%) developed persistent AF (Figure 2). Five (41.7%) patients relapsed with an atypical atrial flutter, while the remaining seven (58.3%) as AF. Out of all patients, one patient (8.3%) suffered symptomatic recurrences and was referred for electrophysiological study and transcatheter redo ablation. Following transcatheter ablation he relapsed with an asymptomatic focal atrial tachycardia. A total of 16 (38%) patients were on antiarrhythmic therapy at follow-up; this percentage did not significantly vary between patients maintaining SR (10, 33%) or suffering AF relapses (6, 50%; *p* = 0.315). Oral anticoagulation was discontinued, instead, in 16 patients (53%) maintaining stable SR at the follow-up end, whereas it was continued in all the patients with documentation of AF relapses.

**Figure 3:**
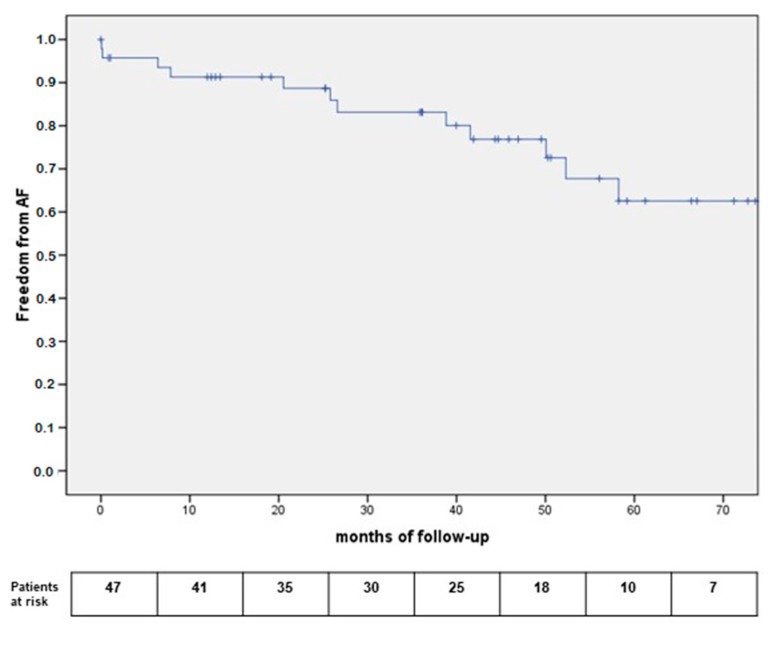
Kaplan Meier curves for freedom from AF relapses.

Freedom from arrhythmias is reported in Figure 3, showing a 91.7% one-year freedom from AF relapses.

During follow-up, four (9.5%) patients suffered from cerebrovascular accidents; two of these subjects had a long-standing persistent AF relapse and were in AF at the time of the event, while the other two were in SR.

Thorough LA morphology and function was assessed at follow-up within 29 patients (69.0%) treated by mitral repair; out of the other 19 patients submitted to mitral valve repair who did not undergo the LA focused echocardiographic evaluation, one (2.1%) was converted to full sternotomy, five (10.4%) died (two of cancer, two of other non-cardiac causes, and only one due to respiratory complications following cardiac arrest), and the remaining 13 patients (27.1%) were contacted only by telephone or fax, owing to problems of access due to long distances between the medical center and the patients’ residences, being our hospital a reference center for surgery.

Among the 29 patients, four (13.8%) had paroxysmal AF, whereas 25 (86.2%) had persistent/long-standing persistent AF at baseline.

In this subset, 22 patients (75.9%) presented a good outcome of mitral repair with a residual mitral regurgitation absent or trivial in 15 subjects (68.2%) and mild in seven (31.8%).

At the moment of the echocardiographic scan, 22 subjects (75.9%) were in SR, while six (20.7%) were in atrial fibrillation/atypical atrial flutter, and one (3.4%) presented an atrial paced rhythm. At univariate analysis the residual mitral regurgitation did not relate to AF relapses (*p* = 0.103).

Table 2 shows details concerning all measurements and functional parameters stratified by heart rhythm at the time of the echo scan. Patients suffering relapses reported more enlarged left atria and more significantly impaired LA function.

Similar trends emerged, as shown in Table 3, if measurements and functional parameters were stratified by heart rhythm during follow-up: 20 (69.0%) SR and nine (31.0%) AF recurrences.

Table 3 points out the markedly enlarged dimensions of the LA, compared to healthy subjects [18], also in patients who maintained SR during the follow-up: the maximum anteroposterior diameter was 51.4 ± 6.2 mm (versus normal values of <41 mm); the maximum indexed volume 60.6 ± 13.1 mL/mq (versus normal values of 22 ± 6 mL/mq); the minimum indexed volume 45.4 ± 10.9 mL/mq (versus normal values of 11 ± 4 mL/mq); the preA indexed volume 51.3 ± 11.6 mL/mq (versus normal values of 15 ± 5 mL/mq); the maximum indexed 3D volume 56.2 ± 13.2 mL/mq (versus normal values of 15–41 mL/mq). However, these same parameter results significantly increased in patients suffering AF relapses at follow-up.

Similarly, SR patients showed a reduction in atrial function when compared to normal values of healthy controls (transmitral A wave velocity 54.7 ± 20.0 cm/s versus 80 ± 20 cm/s [19]; TDI lateral a’ wave 4.6 ± 1.7 cm/s, septal a’ wave 6.0 ± 2.0 cm/s, averaged a’ wave 5.3 ± 1.5 cm/s versus values >7.3 cm/s [17]; LA ejection fraction by areas 17.2 ± 5.0% versus 45% [15]; total emptying fraction by volumes 25.1 ± 7.3% versus normal values ranging from 45% to 65 ± 9% [18,20,21]; active and passive emptying fractions by volumes 11.4 ± 5.4% and 15.5 ± 6.3% versus 46.6 ± 11.7% and 44.3 ± 12.1% [22], respectively; global strain 9.8 ± 3.8% versus 22.9 ± 11.7% [23]). All these parameters, however, were less severely depressed compared to patients with arrhythmia relapses.

At follow-up, patients who suffered from ischemic cerebral events had maximal LA volume/BSA 61 ± 17.8 mL/mq, minimum LA volume/BSA 45.4 ± 5.2 mL/mq, total LA emptying fraction derived from volumes 23.6 ± 13.7%, lateral a’ wave 4.3 ± 0.2 cm/s, and SD of PA-TDI measurements 0.45 ± 0.08. The two patients with strokes in SR showed, instead, extremely enlarged LA volume (>70 mL/mq). One patient suffering a cerebrovascular event despite SR had discontinued oral anticoagulation therapy. The other three patients were on anticoagulation therapy at the time of stroke.

## 4. Discussion

It is reasonable to infer that a lack of recovery of an efficient mechanical atrial activity, even potentially in the presence of SR, facilitates intra-atrial thrombus formation and subsequent systemic thromboembolic phenomena.

Previous studies on surgical AF ablation concomitant to valve surgery reported a highly variable incidence, from 21% to 87%, of both SR restoration and atrial contraction recovery [24,25].

Following the classic LA Maze ablation, it has already been proven that, despite successful SR restoration, there is a progressive loss of LA function, especially within patients affected by rheumatic mitral disease [26].

Following the latest less extensive LA ablation protocols, sparing large areas of the LA, instead, data are controversial. On one side, Loardi et al. analyzed 122 patients: based on transmitral peak A velocity as an atrial contraction index, they proved that 76% of the subjects presented SR and a normal atrial function at three months, and this percentage even increased to 98% after two years [27]. Similarly, Reyes et al. [28], out of 33 patients, highlighted the presence of a transmitral flow in 70% of SR patients at six months follow-up. Analogous results were reported by Manasse et al. [29].

On the other side, in patients with stable SR at six months follow-up, Johansson et al. [30] demonstrated a significant reduction in transmitral A wave velocity in 15 subjects submitted to combined surgery procedure as compared with 14 subjects submitted to mitral valve surgery alone. Boyd et al. suggested that the ablation procedure might have been responsible for the atrial dysfunction observed [31]. In addition, Schiros et al., comparing 35 degenerative mitral regurgitation patients undergoing combined surgery to 51 normal controls by means of cardiac magnetic resonance imaging, asserted that LA volumes and function do not return to normal values at one year distance (in particular, total atrial emptying fraction derived from volumes resulted in 45% pre-operative and 42% at one year post-surgery, versus 54% in controls) [32] supporting the relevant role of the underlying intrinsic disease of the atrial myocardium. Similar results were also reported by Kim et al. [33] within 12 patients: at one month follow-up the LA emptying fraction at computed tomography was 16.8 ± 6.3% (significantly lower than in controls, 47.9 ± 11.2%, *p* < 0.001) despite SR, and atrial contractile function did not improve over the following six months.

Lastly, Compier et al. placed emphasis on the fact that, notwithstanding the limited lesion set during concomitant cardiac surgery, LA compliance, transport function and contraction, decreased after ablation, were not restored in approximately half of the patients with post-procedural SR [34].

The present study highlights the markedly enlarged dimensions and reduced LA function also in patients who maintained SR during the entire follow-up period, compared to data reported in the literature for healthy subjects (see Results section). However, these parameters are more severely depressed, compared to healthy subjects, when patients suffer arrhythmia relapses (Table 3). These results support that concomitant AF cryoablation, by SR maintenance, guarantees an advantage, even if in the setting of deteriorated LAs, in terms of reverse remodeling.

Similar results were found in the HESTER (Has Electrical Sinus Translated into Effective Remodelling?) study [35]: in this experience, adjunct surgical maze was associated with the recovery of LA function but with a mean active LA ejection fraction (ALAEF) lower in maze patients than in control subjects. Providing evidence that function is restored after adjunct maze, potential clinical benefits in reducing thromboembolic and heart failure risk will arise. Determining whether patients can safely stop taking anticoagulants after SR is restored by a maze procedure requires, instead, longer term follow-up and stroke surveillance, beyond those of the HESTER study. In any case, the varying rates of LA functional recovery after maze strongly suggest that, at least, it would be judicious to measure LA function before considering anticoagulation withdrawal.

As stated in a previous study of our group [12] we suggest that, at least within patients with severely enlarged left atrium, previous cardiac surgery and catheter or surgical AF ablation, especially if repeated, the assessment of atrial contractility by transthoracic echocardiography should be performed before discontinuing oral anticoagulants (OAC), also in patients who maintain SR, despite confirmation by serial ECG or Holter monitorings. In our opinion, SR and LA contractility recovery represent two sides of the same coin, and both should be weighted carefully with the objective of selecting patients who could benefit from OAC continuation/discontinuation following an AF ablation.

There is a real need for improvement in patient stratification and personalization of care after AF ablation. Our understanding of which patients should continue anticoagulants for stroke prevention needs to be further refined to reduce the number of patients receiving anticoagulation after a successful AF ablation, and consequently lower the number of hemorrhagic complications, while endorsing the continuation of OAC in patients with a higher risk profile, and prevent thromboembolic events. In this context, we believe echocardiographic assessment of the restoration of an efficient LA contractility should be included among decisional criteria.

Further prospective studies are needed to define the LA dimensions and function cut-off values determining an increase in the risk for thromboembolic complications in patients with restored stable SR. Moreover, quantitative assessment of LA function may have clinical utility in guiding early surgical intervention and concomitant ablation in patients with mitral regurgitation and AF [36,37].

### Study Limitations

This report is an observational and retrospective study. The limited sample size may have influenced the statistical power of the analysis. A comparison with baseline echocardiographic data is not completely feasible because a detailed echocardiography focused on LA morphology and function was not routinely performed before surgery. In addition, echocardiographic follow-up data were not available for the entire cohort of patients. Despite this limitation, we believe our data are representative and of interest. In fact, despite patients lost to follow-up, echocardiographic parameters concerning atrial function in patients followed and with stable SR, after the combined surgical procedure, proved statistically better than in subjects with AF relapse. Being aware that arrhythmia monitoring based on serial 24 h ECG Holter tracings or, even better, implantable recorders would be more accurate, in a mostly persistent AF setting, consideration of any AF relapse, also those present only once during the follow up, should limit event underestimation.

The present study is a starting point; in any case, we are convinced that the concept introduced by the present manuscript may have wider developments in clinical practice.

## 5. Conclusions

Surgical AF cryoablation concomitant to minimally invasive mitral valve repair was determined to be highly effective in maintaining SR and reducing AF burden at long-term follow-up.

The present study highlights the markedly enlarged dimensions of the LA also in patients who maintained SR during the entire follow-up period.

However, at the echocardiographic evaluation, data concerning atrial function in patients with stable SR after the combined surgical procedure are significantly better than in subjects with AF relapse at follow-up. This finding supports the implementation of AF cryoablation concomitant to MIMVS.

## Figures and Tables

**Table 1:** Preoperative characteristics of the study population stratified by AF relapses at follow-up. (AF, atrial fibrillation; AP, antero-posterior; BMI, body mass index; BSA, body surface area; COPD, chronic obstructive pulmonary disease; LS, long-standing; MV, mitral valve; NYHA, New York Heart Association functional class; PAPs, systolic pulmonary artery pressure; SI, supero-inferior; SR, sinus rhythm; TV, tricuspid valve).

	N = 48	AF Relapse (N = 12/42 28.6%)	SR Maintenance (N = 30/42 71.4%)	*p*-Value
Age (years)	67.7 ± 9.8	69.0 ± 6.9	66.7 ± 11.3	0.516
Male gender (N,%)	31 (64.6%)	5 (41.7%)	20 (66.7%)	0.127
NYHA class ≥III (N,%)	31 (64.6%)	10 (83.3%)	17 (56.7%)	0.054
BSA (mq)	1.8 ± 0.19	1.78 ± 0.21	1.82 ± 0.19	0.502
Obesity (BMI > 30) (N,%)	14 (29.2%)	4 (33.3%)	9 (30.0%)	0.619
COPD (N,%)	8 (16.7%)	2 (16.7%)	5 (16.7%)	0.615
Hypertension (N,%)	32 (66.7%)	9 (75.0%)	21 (70.0%)	0.496
Diabetes (N,%)	5 (10.4%)	1 (8.3%)	4 (13.3%)	0.488
Dysthyroidism (N,%)	11 (22.9%)	2 (16.7%)	9 (30.0%)	0.231
Previous cerebrovascular accidents (N,%)	5 (10.4%)	1 (8.3%)	4 (13.3%)	0.488
CHADS2 score	2.29 ± 1.17	2.12 ± 0.93	2.33 ± 1.27	0.542
CHA2DS2VASc score	3.41 ± 1.52	3.47 ± 1.28	3.37 ± 1.63	0.825
Antiarrhythmic therapy at the time of surgery (N,%)	17 (35.4%)	5 (41.7%)	12 (40.0%)	0.473
Type of AF				0.001
Paroxysmal (N,%)	7 (14.6%)	0 (0.0%)	7 (23.3%)	
Persitent/LS persistent (N%)	40 (83.3%)	12 (100%)	22 (73.3%)	
AF duration (days)	1077.6 ± 2052.4	1347.9 ± 2406.3	550.4 ± 849.9	0.127
Left ventricular ejection fraction (%)	57.1 ± 10.5	57.5 ± 10.1	57.6 ± 11.0	0.984
Etiology of MV disease				0.289
Degenerative	32 (66.7%)	7 (58.3%)	22 (73.3%)	
Rheumatic	1 (2.1%)	1 (8.3%)	0 (0.0%)	
Functional	13 (27.1%)	4 (33.3%)	6 (20.0%)	
Left atrial AP diameter (mm)	50.9 ± 10.0	51.1 ± 7.0	51.8 ± 12.1	0.899
Left atrial SI diameter (mm)	65.5 ± 9.1	68.3 ± 10.4	66.6 ± 8.1	0.754
PAPs (mmHg)	43.2 ± 14.1	42.9 ± 12.0	44.1 ± 15.8	0.822

**Table 2:** Echocardiographic parameters and atrial functional evaluations, expressed as mean of the total population and stratified on the basis of presenting rhythm during echocardiography. (AF, atrial fibrillation; AP, antero-posterior; BSA, body surface area; DT, deceleration time; EF, ejection fraction; LA, left atrial; LV, left ventricular; PAPs, systolic pulmonary arterial pressure; RA, right atrial; SI, supero-inferior; SR, sinus rhythm).

	N = 29	SR (N = 22/29)	AF (N = 6/29)	*p*-Value
Max LA AP diameter (mm)	53.4 ± 6.7	51.5 ± 5.9	59.8 ± 6.0	0.018
Min LA AP diameter (mm)	46.7 ± 7.2	44.4 ± 6.0	55.0 ± 5.1	0.002
Total LA emptying fraction derived from AP diameters (%)	12.7 ± 4.4	14.1 ± 4.1	8.0 ± 1.6	0.006
Max LA SI diameter (mm)	68.3 ± 5.4	67.2 ± 4.6	74.0 ± 3.7	0.003
Max LA area (cmq)	31.8 ± 5.2	30.4 ± 4.3	37.3 ± 5.5	0.010
Min LA area (cmq)	27.0 ± 5.1	25.3 ± 3.8	33.4 ± 4.8	0.001
LA ejection fraction derived from areas (%)	15.5 ± 5.4	16.7 ± 5.3	10.5 ± 2.8	0.028
Max LA volume (mL)	120.4 ± 32.6	111.5 ± 25.9	152.8 ± 39.0	0.016
Max LA volume/BSA (mL/mq)	65.6 ± 15.8	60.9 ± 12.5	80.7 ± 18.2	0.010
Min LA volume (mL)	93.5 ± 28.7	84.4 ± 21.1	127.7 ± 30.9	0.002
Min LA volume/BSA (mL/mq)	60.0 ± 14.5	46.2 ± 11.2	67.5 ± 14.4	0.002
3D LA volume (mL)	111.6 ± 38.2	100.2 ± 25.5	158.1 ± 51.7	0.005
3D LA volume/BSA (mL/mq)	61.1 ± 18.9	54.9 ± 12.4	84.0 ± 24.9	0.003
Total LA emptying fraction derived from volumes (%)	22.7 ± 8.0	24.4 ± 7.9	16.0 ± 5.7	0.061
Global longitudinal LA strain (%)	8.5 ± 3.8	9.7 ± 3.6	5.2 ± 1.7	0.013
E wave (cm/s)	137.2 ± 23.7	136.4 ± 25.8	143.5 ± 14.4	0.552
A wave (cm/s)	56.2 ± 19.8	57.1 ± 19.8		0.286
Inferior septal a’ wave (cm/s)	5.8 ± 2.0	5.9 ± 2.0		0.508
Lateral a’ wave (cm/s)	4.5 ± 1.6	4.6 ± 1.6		0.356
Averaged a’ wave (cm/s)	5.2 ± 1.5	5.2 ± 1.5		0.352
P-lateral a’ wave interval (ms)	110.6 ± 31.9	110.3 ± 32.7		0.866
LV EF (%)	57.3 ± 8.0	57.9 ± 6.8	53.8 ± 11.7	0.352
PAPs (mmHg)	37.7 ± 9.4	37.0 ± 9.6	38.2 ± 9.4	0.479

**Table 3:** Echocardiographic parameters and atrial functional evaluations, stratified according to SR maintenance and type of AF recurrence during follow-up. (AF, atrial fibrillation; AP, antero-posterior; BSA, body surface area; DT, deceleration time; EF, ejection fraction; LA, left atrial; LS, long-standing; LV, left ventricular; PAPs, systolic pulmonary arterial pressure; RA, right atrial; SD, standard deviation; SI, supero-inferior; SR, sinus rhythm).

	Stable SR (N = 20/29)	AF Relapse (N = 9/29)	Paroxysmal AF Relapse (N = 3/29)	Persistent/LS Persistent AF Relapse (N = 6/29)	*p*-Value
Max LA AP diameter (mm)	51.4 ± 6.2	57.8 ± 5.9	53.7 ± 3.2	59.8 ± 6.0	0.019
Min LA AP diameter (mm)	44.3 ± 6.3	52.2 ± 6.0	46.7 ± 3.2	55.0 ± 5.1	0.002
P wave LA AP diameter (mm)	47.5 ± 6.1		49.7 ± 4.2		0.565
Active LA emptying fraction derived from AP diameters (%)	7.0 ± 3.2		5.9 ± 3.0		0.601
Passive LA emptying fraction derived from AP diameters (%)	7.7 ± 3.4		7.5 ± 2.3		0.945
Total LA emptying fraction derived from AP diameters (%)	14.1 ± 4.2	9.7 ± 3.0	13.1 ± 1.9	8.0 ± 1.6	0.006
Max LA SI diameter (mm)	67.0 ± 4.9	71.4 ± 5.2	66.3 ± 4.0	74.0 ± 3.7	0.009
Max LA area (cmq)	30.1 ± 4.3	35.6 ± 5.3	32.2 ± 3.1	37.3 ± 5.5	0.007
Min LA area (cmq)	24.9 ± 3.8	31.4 ± 4.9	27.5 ± 2.2	33.4 ± 4.8	<0.001
P wave LA area (cmq)	27.0 ± 4.0		28.9 ± 2.2		0.430
LA ejection fraction derived from areas (%)	17.2 ± 5.0	11.8 ± 4.6	14.3 ± 7.2	10.5 ± 2.8	0.022
Max LA volume (mL)	109.9 ± 26.4	143.8 ± 34.4	125.7 ± 13.6	152.8 ± 39.0	0.012
Max LA volume/BSA (mL/mq)	60.6 ± 13.1	76.8 ± 16.2	69.0 ± 9.1	80.7 ± 18.2	0.016
Min LA volume (mL)	82.4 ± 21.0	118.2 ± 28.7	99.3 ± 10.2	127.7 ± 30.9	0.001
Min LA volume/BSA (mL/mq)	45.4 ± 10.9	63.3 ± 14.2	55.0 ± 11.6	67.5 ± 14.4	0.002
P wave LA volume (mL)	92.9 ± 22.7		104.3 ± 8.1		0.402
P wave LA volume/BSA (mL/mq)	51.3 ± 11.6		57.6 ± 10.4		0.380
3D LA volume (mL)	101.6 ± 26.6	134.0 ± 51.6	93.9 ± 11.2	158.1 ± 51.7	0.005
3D LA volume/BSA (mL/mq)	56.2 ± 13.2	71.9 ± 25.6	51.7 ± 8.4	84.0 ± 24.9	0.005
Active LA emptying fraction derived from volumes (%)	11.4 ± 5.4		4.9 ± 3.0		0.057
Passive LA emptying fraction derived from volumes (%)	15.5 ± 6.3		16.5 ± 9.0		0.809
Total LA emptying fraction derived from volumes (%)	25.1 ± 7.3	17.5 ± 7.4	20.4 ± 10.9	16.0 ± 5.7	0.039
Global longitudinal LA strain (%)	9.8 ± 3.8	5.8 ± 1.9	7.0 ± 2.0	5.2 ± 1.7	0.021
E wave (cm/s)	131.4 ± 24.2	150.0 ± 17.6	163.0 ± 18.2	143.5 ± 14.4	0.070
A wave (cm/s)	54.7 ± 20.0		66.1 ± 19.5		0.364
Inferior septal a’ wave (cm/s)	6.0 ± 2.0		4.9 ± 2.2		0.409
Lateral a’ wave (cm/s)	4.6 ± 1.7		3.6 ± 0.8		0.306
Averaged a’ wave (cm/s)	5.3 ± 1.5		4.3 ± 1.5		0.274
P-lateral a’ wave interval (ms)	110.8 ± 28.9		109.0 ± 57.3		0.930
SD P-a’ wave intervals	0.56 ± 0.31		0.63 ± 0.47		0.736
LV EF (%)	58.0 ± 7.2	55.8 ± 9.8	59.7 ± 3.5	53.8 ± 11.7	0.484
PAPs (mmHg)	36.2 ± 9.3	40.7 ± 9.5	45.7 ± 9.0	38.2 ± 9.4	0.284

## References

[1] Chua Y.L., Schaff H.V., Orszulak T.A., Morris J.J. (1994). Outcome of mitral valve repair in patients with preoperative atrial fibrillation. Should the maze procedure be combined with mitral valvuloplasty?. J. Thorac. Cardiovasc. Surg..

[2] Scarsoglio S., Saglietto A., Gaita F., Ridolfi L., Anselmino M. (2016). Computational fluid dynamics modelling of left valvular heart diseases during atrial fibrillation. PeerJ.

[3] Gaita F., Ebrille E., Scaglione M., Caponi D., Garberoglio L., Vivalda L., Barbone A., Gallotti R. (2013). Very long-term results of surgical and transcatheter ablation of long-standing persistent atrial fibrillation. Ann. Thorac. Surg..

[4] Ngaage D.L., Schaff H.V., Mullany C.J., Barnes S., Dearani J.A., Daly R.C., Orszulak T.A., Sundt T.M. (2007). Influence of preoperative atrial fibrillation on late results of mitral repair: Is concomitant ablation justified?. Ann. Thorac. Surg..

[5] Chitwood W.R., Wixon C.L., Elbeery J.R., Moran J.F., Chapman W.H., Lust R.M. (1997). Video-assisted minimally invasive mitral valve surgery. J. Thorac. Cardiovasc. Surg..

[6] Marchetto G., Anselmino M., Rovera C., Mancuso S., Ricci D., Antolini M., Morello M., Gaita F., Rinaldi M. (2016). Results of cryoablation for atrial fibrillation concomitant with video-assisted minimally invasive mitral valve surgery. Semin. Thorac. Cardiovasc. Surg..

[7] Schaff H.V. (2015). Surgical ablation of atrial fibrillation—When, why, and how?. N. Engl. J. Med..

[8] Blomström-Lundqvist C., Johansson B., Berglin E., Nilsson L., Jensen S.M., Thelin S., Holmgren A., Edvardsson N., Källner G., Blomström P. (2007). A randomized doubleblind study of epicardial left atrial cryoablation for permanent atrial fibrillation in patients undergoing mitral valve surgery: The SWEDish Multicentre Atrial Fibrillation study (SWEDMAF). Eur. Heart J..

[9] Phan K., Xie A., Tian D.H., Shaikhrezai K., Yan T.D. (2014). Systematic review and meta-analysis of surgical ablation for atrial fibrillation during mitral valve surgery. Ann. Cardiothorac. Surg..

[10] Stulak J.M., Schaff H.V. (2016). The cardiac surgeon as electrophysiologist. J. Thorac. Cardiovasc. Surg..

[11] Jeevanantham V., Ntim W., Navaneethan S.D., Shah S., Johnson A.C., Hall B., Shah A., Hundley W.G., Daubert J.P., Fitzgerald D. (2010). Meta-analysis of the effect of radiofrequency catheter ablation on left atrial size, volumes and function in patients with atrial fibrillation. Am. J. Cardiol..

[12] Anselmino M., Rovera C., Marchetto G., Ferraris F., Castagno D., Gaita F. (2017). Anticoagulant cessation following atrial fibrillation ablation: Limits of the ECG-guided approach. Expert Rev. Cardiovasc. Ther..

[13] Barbero C., Marchetto G., Ricci D., El Qarra S., Attisani M., Filippini C., Boffini M., Rinaldi M. (2016). Right minithoracotomy for mitral valve surgery: Impact of tailored strategies on early outcome. Ann. Thorac. Surg..

[14] Ad N., Holmes S.D., Lamont D., Shuman D.J. (2017). Left-sided surgical ablation for patients with atrial fibrillation who are undergoing concomitant cardiac surgical procedures. Ann. Thorac. Surg..

[15] Gutman J., Wang Y.S., Wahr D., Schiller N.B. (1983). Normal left atrial function determined by 2-dimensional echocardiography. Am. J. Cardiol..

[16] Manning W.J., Leeman D.E., Gotch P.J., Come P.C. (1989). Pulsed Doppler evaluation of atrial mechanical function after electrical cardioversion of atrial fibrillation. J. Am. Coll. Cardiol..

[17] Wang M., Yip G.W., Wang A.Y., Zhang Y., Ho P.Y., Tse M.K., Lam P.K., Sanderson J.E. (2003). Peak early diastolic mitral annulus velocity by tissue Doppler imaging adds independent and incremental prognostic value. J. Am. Coll. Cardiol..

[18] Lupu S., Mitre A., Dobreanu D. (2014). Left atrium function assessment by echocardiography—physiological and clinical implications. Med. Ultrason..

[19] Vasan R.S., Larson M.G., Levy D., Galderisi M., Wolf P.A., Benjamin E.J. (2003). Doppler transmitral flow indexes and risk of atrial fibrillation (the Framingham Heart Study). Am. J. Cardiol..

[20] Le Bihan D.C., Della Togna D.J., Barretto R.B., Assef J.E., Machado L.R., Ramos A.I., Abdulmassih Neto C., Moisés V.A., Sousa A.G., Campos O. (2015). Early improvement in left atrial remodeling and function after mitral valve repair or replacement in organic symptomatic mitral regurgitation assessed by three-dimensional echocardiography. Echocardiography.

[21] Ring L., Rana B.S., Wells F.C., Kydd A.C., Dutka D.P. (2014). Atrial function as a guide to timing of intervention in mitral valve prolapse with mitral regurgitation. JACC Cardiovasc. Imaging.

[22] Leischik R., Littwitz H., Dworrak B., Garg P., Zhu M., Sahn D.J., Horlitz M. (2015). Echocardiographic evaluation of left atrial mechanics: Function, history, novel techniques, advantages, and pitfalls. Biomed. Res. Int..

[23] Hammerstingl C., Schwekendiek M., Momcilovic D., Schueler R., Sinning J.M., Schrickel J.W., Mittmann-Braun E., Nickenig G., Lickfett L. (2012). Left atrial deformation imaging with ultrasound based two-dimensional speckle-tracking predicts the rate of recurrence of paroxysmal and persistent atrial fibrillation after successful ablation procedures. J. Cardiovasc. Electrophysiol..

[24] Feinberg M.S., Waggoner A.D., Kater K.M., Cox J.L., Lindsay B.D., Perez J.E. (1994). Restoration of atrial function after the maze procedure for patients with atrial fibrillation: Assessment by Doppler echocardiography. Circulation.

[25] Yuda S., Nakatani S., Isobe F., Kosakai Y., Miyatake K. (1998). Comparative efficacy of the maze procedure for restoration of atrial contraction in patients with and without giant left atrium associated with mitral valve disease. J. Am. Coll. Cardiol..

[26] Kim H.W., Moon M.H., Jo K.H., Song H., Lee J.W. (2015). Left atrial and left ventricular diastolic function after the maze procedure for atrial fibrillation in mitral valve disease: Degenerative versus rheumatic. Indian J. Surg..

[27] Loardi C., Alamanni F., Galli C., Naliato M., Veglia F., Zanobini M., Pepi M. (2015). Surgical treatment of concomitant atrial fibrillation: Focus onto atrial contractility. Biomed. Res. Int..

[28] Reyes G., Benedicto A., Bustamante J., Sarraj A., Nuche J.M., Alvarez P., Duarte J. (2009). Restoration of atrial contractility after surgical cryoablation: Clinical, electrical and mechanical results. Interact. Cardiovasc. Thorac. Surg..

[29] Manasse E., Gaita F., Ghiselli S., Barbone A., Garberoglio L., Citterio E., Ornaghi D., Gallotti R. (2003). Cryoablation of the left posterior atrial wall: 95 patients and 3 years of mean follow-up. Eur. J. Cardiothorac. Surg..

[30] Johansson B., Bech-Hanssen O., Berglin E., Blomström P., Holmgren A., Jensen S.M., Källner G., Nilsson L., Thelin S., Karlsson T. (2012). Atrial function after left atrial epicardial cryoablation for atrial fibrillation in patients undergoing mitral valve surgery. J. Interv. Card. Electrophysiol..

[31] Boyd A.C., Schiller N.B., Ross D.L., Thomas L. (2009). Differential recovery of regional atrial contraction after restoration of sinus rhythm after intraoperative linear radiofrequency ablation for atrial fibrillation. Am. J. Cardiol..

[32] Schiros C.G., Ahmed M.I., McGiffin D.C., Zhang X., Lloyd S.G., Aban I., Denney T.S., Dell’Italia L.J., Gupta H. (2015). Mitral annular kinetics, left atrial, and left ventricular diastolic function post mitral valve repair in degenerative mitral regurgitation. Front. Cardiovasc. Med..

[33] Kim J.B., Yang D.H., Kang J.W., Jung S.H., Choo S.J., Chung C.H., Song J.K., Lee J.W. (2015). Left atrial function following surgical ablation of atrial fibrillation: Prospective evaluation using dual-source cardiac computed tomography. Yonsei Med. J..

[34] Compier M.G., Tops L.F., Braun J., Zeppenfeld K., Klautz R.J., Schalij M.J., Trines S.A. (2017). Limited left atrial surgical ablation effectively treats atrial fibrillation but decreases left atrial function. Europace.

[35] Abu-Omar Y., Thorpe B.S., Freeman C., Mills C., Stoneman V.E.A., Gopalan D., Rana B., Spyt T.J., Sharples L.D., Nashef S.A.M. (2017). Recovery of left atrial contractile function after maze surgery in persistent longstanding atrial fibrillation. JACC.

[36] Ring L., Abu-Omar Y., Kaye N., Rana B.S., Watson W., Dutka D.P., Vassiliou V.S. (2018). Left atrial function is associated with earlier need for cardiac surgery in moderate to severe mitral regurgitation: Usefulness in targeting for early surgery. J. Am. Soc. Echocardiogr..

[37] Anselmino M., Gaita F., Saglietto A. (2017). Effectiveness of catheter ablation of atrial fibrillation: Are we at the dawn of a new era?. J. Thorac. Dis..

